# The efficacy and safety of probiotics in the adjuvant treatment of psoriasis: a systematic review and meta-analysis of randomized controlled trials

**DOI:** 10.3389/fmed.2024.1448626

**Published:** 2024-09-06

**Authors:** Yiran Zhu, Fan Xu, Hao Chen, Quanhui Zheng

**Affiliations:** ^1^School of Basic Medical Sciences, North China University of Science and Technology, Tangshan, China; ^2^Hebei Key Laboratory for Chronic Diseases, Tangshan Key Laboratory for Preclinical and Basic Research on Chronic Diseases, School of Basic Medical Sciences, North China University of Science and Technology, Tangshan, China

**Keywords:** psoriasis, probiotics, meta-analysis, randomized controlled trial, placebo

## Abstract

**Background:**

It has been reported that the imbalance of gut microbiota is involved in the pathogenesis of psoriasis. We retrieved randomized placebo-controlled trials to evaluate the efficacy and safety of probiotic administration in the treatment of psoriasis.

**Methods:**

The outcomes were changes in Psoriasis Area and Severity Index (PASI), Dermatology Life Quality Index (DLQI), and serum inflammatory indicators after treatment, and adverse events (AEs). Risk ratios (RRs) and mean differences (MDs) were calculated using random or fixed effects model.

**Results:**

Seven qualified studies were identified in our study. The pooled percentage of patients with ≥75% reduction from baseline in PASI was higher in the probiotic group than that in the placebo group (33.57% vs. 23.61%; RR 1.40, 95% CI 0.98–1.98, *p* = 0.06). Compared with the placebo group, the PASI (MD −3.09, 95% CI −5.04 to −0.74, *p* = 0.01) and CRP level (MD −2.36, 95% CI −2.77 to −1.95, *p* < 0.0001) were significantly reduced in the probiotic group. There was no significant difference in DLQI (MD −1.45, 95% CI −6.72 to 3.82, *p* = 0.59) and AEs (RR 0.68, 95% CI 0.37–1.25, *p* = 0.22) between the two groups.

**Conclusion:**

Oral administration of probiotics can improve psoriasis; however, large randomized controlled trials are needed to support this conclusion.

**Systematic review registration:**

PROSPERO, identifier CRD42024506286, https://www.crd.york.ac.uk/prospero/display_record.php?ID=CRD42024506286.

## Introduction

Psoriasis is a chronic inflammatory skin disease associated with environmental factors, hereditary susceptibility and immune disorders. In addition to skin involvement, some patients may have arthritis, inflammatory bowel disease, metabolic syndrome, and an increased risk of cardiovascular disease (1). Patients with psoriasis have a tendency for chronic recurrence, which seriously affects their quality of life. T lymphocytes are the key factors in the occurrence and progression of psoriasis. Previously, T helper (Th)1, currently Th17, is believed to be the major effector in the pathogenesis, with increased expression of inflammatory cytokines such as IL-1, IL-2, IL-6, IL-8, IL-12, IL-17, IL-23 and TNF-α, leading to the hyperproliferation and aberrant differentiation of epidermal keratinocytes (2).

The gut microbiota, which consists of trillions of microbial communities, is involved in many local and systemic processes and is recognized as a virtual organ closely associated with the health of the host. Intestinal epithelial cells, immune cells, and the flora interact to form specific immune responses to antigens (3). Some commensal bacteria, such as *Lactobacillus*, *Bifidobacterium*, a few *Escherichia coli* strains, and *Akkermansia muciniphila*, can maintain the integrity of the intestinal mucosa and contribute to a healthy immune system (3, 4). When the relationship between the gut microbiota and the mucosal immunity is impaired, there may be subsequent disturbance of mucosal immune tolerance, leading to skin diseases (5).

Several studies investigated the composition of the intestinal microbiota in psoriasis subjects and found that the alpha diversity and beta diversity of the gut microbiota were significantly reduced compared with healthy control (6–8). A reduction in the abundance of potentially beneficial microbes, such as *Bacteroides genus*, *Proteobacteria*, and *Akkermansia muciniphila*, was found in patients with psoriasis (9, 10). The transfer of intestinal microbiota from the K14-VEGF transgenic mouse model with severe psoriasis skin phenotype increased the abundance of *Prevotella* and decreased the abundance of *Parabacteroides distasonis* in the colon, resulting in aggravation of psoriasiform skin inflammation and augmentation of Th17 infiltration and differentiation in mice with mild symptoms (11). These results suggest that by regulating gut microbiota with probiotic supplements, a new approach for psoriasis treatment may be developed.

A recent animal study showed that *Bifidobacterium breve CCFM683* administration can dose-dependently ameliorate psoriasis by restoring microflora, maintaining intestinal epidermal barrier function, and reducing pro-inflammatory cytokines (12). Several randomized double-blind placebo-controlled studies have evaluated the efficacy of probiotic supplements for the treatment of psoriasis. Moludi et al. reported that the severity of psoriasis and the quality of life were significantly improved in patients treated with a multi-strain probiotic supplement for 8 weeks, with a considerable reduction in serum levels of C-reactive protein (CRP), IL-6, IL-1β and LPS (13). However, Suriano et al. administered *Lactobacillus rhamnosus* to patients with psoriasis for 6 months, and showed no statistically significant differences in the changes of Psoriasis Area and Severity Index (PASI) and Dermatology Life Quality Index (DLQI) from baseline between the probiotic and control groups after treatment (14). A meta-analysis that included only 2 randomized controlled trials (RCTs) showed that oral probiotics may have a positive effect on alleviating the clinical symptoms in patients with psoriasis, but the summarized mean difference (MD) of PASI between the probiotic and placebo groups was not statistically significant (7). In addition, the two included RCTs differed in how PASI improved after treatment, which may bias the combined MD values (15). Thus, the quality of the evidence in the meta-analysis was very low. An updated meta-analysis is necessary to clarify the therapeutic effect of probiotics on psoriasis.

The aims of this meta-analysis were to include randomized, double-blind, placebo-controlled studies to evaluate the efficacy and safety of oral probiotics in the treatment of psoriasis and to provide a basis for future clinical decision-making.

## Materials and methods

This systematic review and meta-analysis was strictly conducted in accordance with the Preferred Reporting Items for Systematic Reviews and Meta-Analyses (PRISMA) guidelines (Supplementary Table S1). The protocol was originally registered in PROSPERO (CRD42024506286).

### Inclusion and exclusion criteria

The trials included in this meta-analysis were all RCTs, which were selected in accordance with the PICO criteria: (1) participants: All participants were patients diagnosed with psoriasis older than 18 years old, in whom symptoms were graded using PASI or other diagnostic criteria for psoriasis. (2) Intervention and comparison: probiotics were used as a treatment in experimental groups that did not limit in species, characteristics, etc. Placebo was used in the control group. (3) Outcomes: PASI and DLQI, the serum levels of CRP and interleukin-6, and adverse events (AEs). Studies were excluded if they were case reports, meta-analyses, reviews, animal studies, non-RCT studies or non-placebo-controlled studies, literature with incomplete data, or duplicate publications.

### Literature search strategy

Four databases PubMed, Medline, Embase, and Cochrane Central Register, were searched in English until December 2023. PubMed search strategy: “(((((((random* controlled trial) OR (controlled clinical trial*)) OR (randomized)) OR (placebo)) OR (random*)) OR (trial*)) AND (probiotic*)) AND (((((Psoriasis) OR (Pustulosis of Palms and Soles)) OR (Pustulosis Palmaris et Plantaris)) OR (Palmoplantaris Pustulosis)) OR (Pustular Psoriasis of Palms and Soles)).”

### Data extraction

The data from the included studies were independently extracted by two researchers using a predesigned form including first author, year of publication, study period, country, numbers of patients in each group, basic characteristics of subjects, inclusion and exclusion criteria, probiotic strains, duration of treatment, and outcome indicators (percentage of patients with ≥75% reduction from baseline in PASI, PASI score, DLQI score, levels of CRP and IL-6, AEs). If the standard deviation was unavailable from the original study, it was calculated from other measures of dispersion (e.g., standard error or interquartile range) as reported. If the mean and standard deviation of parameter changes before and after treatment were not directly available (e.g., the study only reported the mean and standard deviation of individual time points, or the mean and standard error, or interquartile ranges), the mean and standard deviation were calculated based on the relevant data of baseline and relevant time points. If there was any disagreement, a third reviewer was consulted and discussed to reach a conclusion.

### Risk of bias assessment

Risk of bias assessment was conducted using the Cochrane Risk of Bias Evaluation standard. All studies were evaluated according to the following standards: random sequence generation, allocation concealment, blinding of patients and personnel, incomplete outcome data, selective reporting, and other bias (16). Traffic light plots were created for domain-level judgements of each individual result, and weighted bar plots were generated to present the distribution of risk-of-bias judgements within each bias domain. We considered studies with a score of 3 or more to be of high quality. The authors assessed the quality of each study separately and reached a consensus on the included studies.

### Statistical analysis

RevMan 5.3 software was used to conduct the statistical analysis. Risk ratios (RRs) and the corresponding 95% confidence intervals (CIs) were used for the percentage of patients with ≥75% reduction from baseline in PASI and AEs after treatment. As the results for the changes in PASI and DLQI from baseline after treatment, and the levels of CRP and IL-6 were continuous data, the mean difference (MD) and 95% CI were calculated for statistical analyses. If the MD value was not available, it was calculated using the Evidence-based medicine data extraction and transformation table or an Internet resource.^1^ Heterogeneity in the combined results was assessed using the I^2^ statistic and χ^2^ test. Obvious heterogeneity was indicated when *p* < 0.10, and I^2^ > 50% in the χ^2^ test. If heterogeneity was obvious, the random-effects model was used. If heterogeneity was low, a fixed model was used. Sensitivity analyses were conducted to explore the extent to which our results were altered by excluding studies one-by-one. Subgroup analysis was performed with regard to the treatment duration. Because of the small number of trials included in this meta-analysis, publication bias analysis was not performed (17). Statistical significance was defined as *p* < 0.05.

## Results

### Search results and characteristics of the included studies

**Figure 1:**
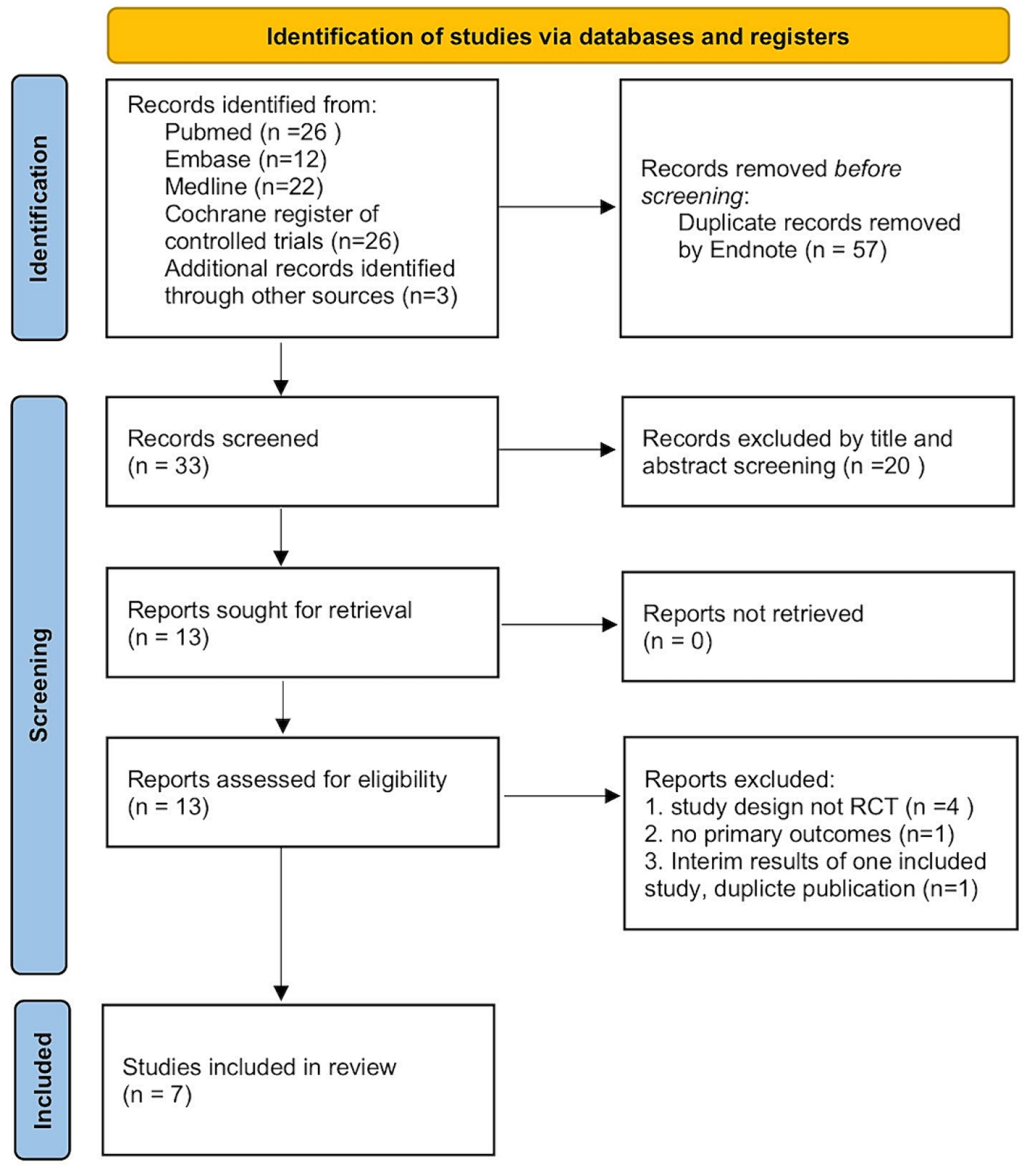
Flow diagram of the included studies.

A total of 86 articles were screened initially through database retrieval, and 3 articles were selected through other sources. After duplicate records were removed and excluded by title and abstract, 13 articles were collected. After carefully reading the full text and comparing the selection criteria, 7 qualified RCTs containing 400 patients (treatment group 198 patients; controlled group 202 patients) were finally included in our meta-analysis (Figure 1) (13, 14, 18–22). Two studies conducted by the same team with different registration numbers were both included in our study (13, 19). The main characteristics of the enrolled studies were listed in Table 1.

**Table 1:** Characteristics of the included studies.

First author	Year	Country	Study design	Inclusion criteria	Exclusion criteria	No. of patients	Basic treatment	Treatment group	Control group	Duration	Outcomes
Navarro-Lopez et al. (18)	2019	Spain	Randomized, double-blind, placebo-controlled	18–70 years old, plaque psoriasis at least one year prior to study, mild or moderate severity (PASI>6)	Patients exposed to systemic corticosteroids, methotrexate, cyclosporine, biologic drugs in the previous 3 months, antibiotics in the previous 2 weeks, signs of bacterial infection, liver disease with Child-Pugh C, chronic renal insufficiency, chronic endocrine, respiratory, neurological or moderate to severe cardiovascular disease, concomitant skin disease	88	Patients with PASI≥6 were prescribed betamethasone dipropionate together with calcipotriol once a day, patients with PASI<6 were prescribed mometasone furoate	*Bifidobacterium longum* CECT 7347, *B. lactis* CECT 8145, *Lactobacillus rhamnosus* CECT 8361, 1 × 10^9^ CFU per capsule, 1 capsule daily	A capsule containing only maltodextrin, matched for size, shape, and volume of contents	12 wks	PASI, PGA, TNF-α, IFN-γ, IL-1β, IL-6, IL-12, IL-23
Moludi (19)	2021	Iran	Randomized double-blind, placebo-controlled	18–50 years old, recent psoriasis	Patients used any antibiotic or probiotics in last 8 weeks, malignancy, inflammatory bowel disease, cardiovascular disease, liver disease and inflammatory disease.	50	N/A	*Lactobacillus acidophilus*, *Bifidobacterium bifidum*, *Bifidobacterium lactis*, Bifidobacterium langum, 1.8 × 10^9^ CFU per capsule, 1 capsule twice a day	Maltodextrin capsules with the same size and shape	8 wks	DLQI, PSS, PASI, BDI-II, hs-CRP, IL-6, MDA, TAC
Moludi (13)	2022	Iran	Randomized double-blind, placebo-controlled	Patients being diagnosed with psoriasis or at least 6 months, and never treated with systemic disease-modifying anti-rheumatic drugs	Patients refused to participate or use any current unusual diet (macrobiotic diet), probiotics and antibiotic supplements, or an autoimmune disease, like inflammatory bowel disease or inflammatory arthritis	46	Take routine drugs, any antioxidants was forbidden	*Lactobacillus acidophilus*, *Bifidobacterium bifidum*, *Bifidobacterium lactis*, Bifidobacterium langum, 1.6 × 10^9^ CFU/g, twice a day	Received maltodextrin capsules		PASI, QOL, hs-CRP, IL-6, IL-1β, LPS
Akbarzadeh (20)	2022	Iran	Randomized double-blind, placebo-controlled	Psoriasis aged 18–60 years and a lack of history of consumption of probiotics and drugs 1–6 weeks before the beginning of the experiment, PASI ≥2%	Diabetic and immunosuppressant patients and patients with a history of immunosuppressing drug consumption	52	Weak corticosteroid or hydrocortisone topically	Lactocare^®^ capsules contain 12 strains of probiotic species including Lactobacillus strains, Bifidobacteria strains, *Streptococcus thermophilus*, plus Fructo-oligosaccharides as the prebiotic (1 × 10^9^ CFU/capsules)	Placebo	12 wks	PASI, DLQI, VAS
Groeger (21)	2013	Ireland	Randomized, double-blind placebo-controlled	18–60 years mild to moderate chronic plaque psoriasis with a PASI <16	Pregnant or breast feeding females, individuals with lactose intolerance or immunodeficiency, individuals who had undergone any abdominal surgery and those with a psychiatric illness or significant hepatic, renal disease, receiving immunosuppressant therapy or probiotics.	26	N/A	1 × 10^10^ CFU viable *Bifidobacterium infantis* 35,264	5 g Maltodextran as placebo	8 wks	CRP, TNF-α, IL-6
Suriano (14)	2023	Brazil	Randomized, double-blind placebo-controlled	18 years old patients with plaque psoriasis	Pregnant females, patients having other skin diseases, neoplasms nor systemic inflammatory diseases (such as Crohn’s disease and inflammatory bowel disease)	103	Standard-of-care	*Lactobacillus rhamnosus* 6×10 CFU/ml, drink 5 mL daily	Placebo	6 mths	PASI, DLQI
Gilli (22)	2022	Brazil	Randomized, double-blind placebo-controlled	Patients older than 18 years, clinically diagnosed with psoriasis	Patients started systemic medication in the 3 months prior to the study. During the study, if it was necessary to change the psoriasis medication, the patient would be excluded	35	Regular use of standard medication including topical therapy (Clobetasol, Betamethasone Dipropionate, Calcipotriol) combined with systemic treatment (Methotrexate and Secukinumab, Ustekinumab or Adalimumab)	1 capsule of 5 × 10^9^ CFU/g of L rhamnosus Lr-G14/day for 60 days	Placebo	60 days	PASI, DLQI, BSA, IL-23, IL17

PASI, Psoriasis Area and Severity Index; PGA, Physician Global Assessment; DLQI, Dermatology Life Quality Index; QOL, quality of life; PSS, Psoriasis Symptom Scale; BSA, Body Surface Area; BDI-II, Beck Depression Inventory-II; VAS, Visual Analogue Scale; CRP, C-reactive protein; TNF-α, tumor necrosis factor α; IL-6, interleukin-6; MDA, malondialdehyde; TAC, total antioxidant capacity; CFU, colony-forming units.

### Quality assessment

**Figure 2:**
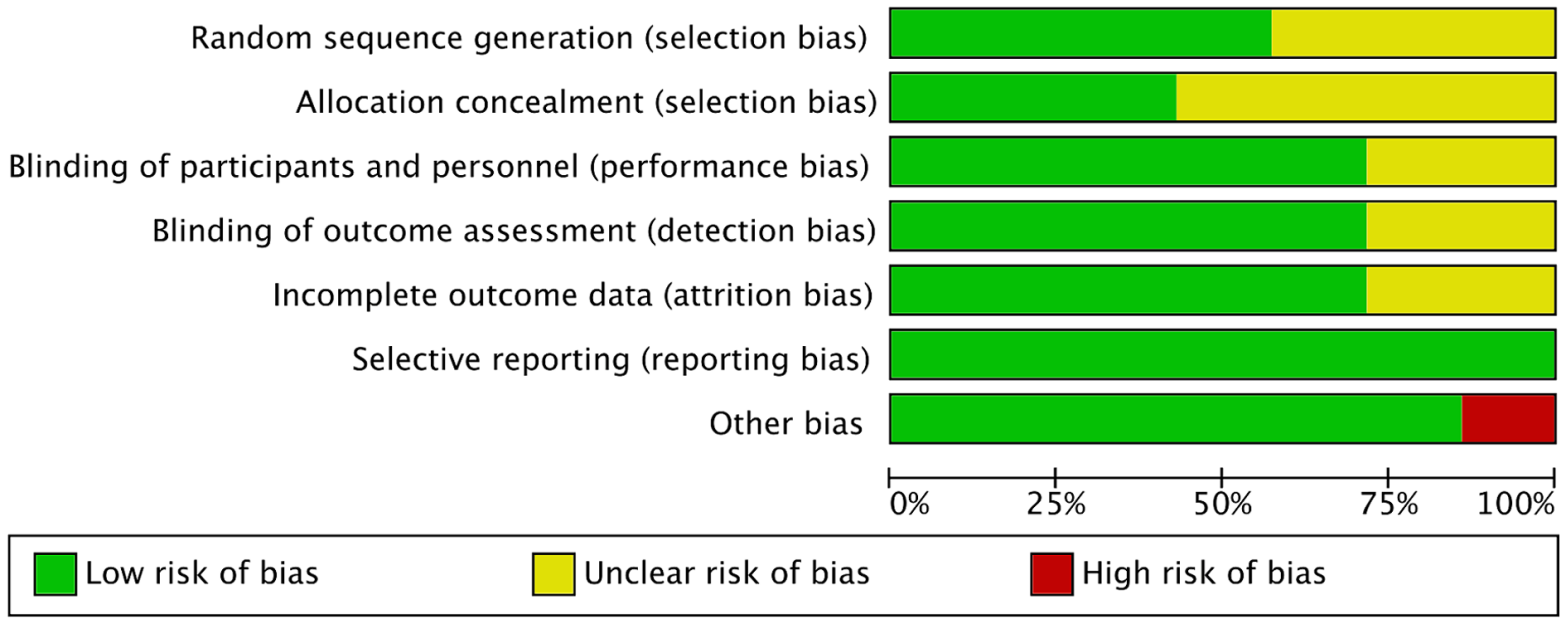
Risk of bias of all included studies.

**Figure 3:**
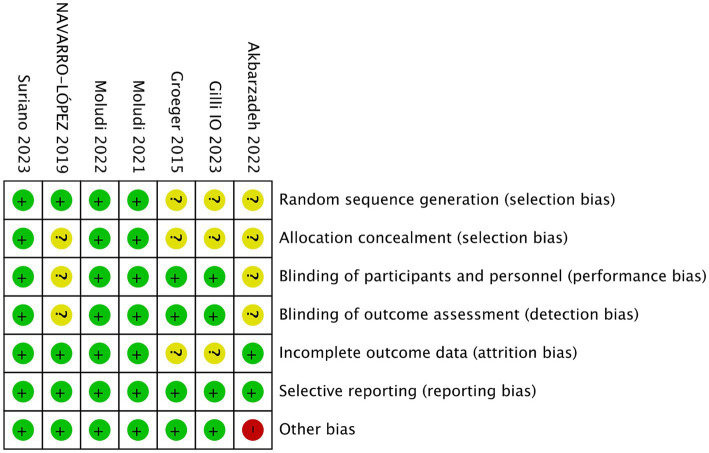
Risk of bias for each included study.

Figures 2, 3 showed the risk of bias of the included RCTs as judged by two reviewers. Quality assessment of all included studies was summarized in Supplementary Table S2.

### Random sequence generation

Three studies did not provide sufficient information about the generation process of random sequence, and could not be judged as low or high risk (20–22). The other 4 studies reported that randomization was performed using computer-generated randomized numbers, and were rated as low risk of bias (13, 18, 19, 22).

### Allocation concealment

Four studies did not describe whether allocation concealment was performed, the information was unclear, and therefore were assessed as unclear risk of bias (18, 21, 22). The other three studies used sealed envelopes, the same looking drugs, or pharmacy-controlled randomization, and were therefore rated as low risk (13, 14, 19).

### Blinding

Akbarzadeh et al. (20) and Navarro-Lopez et al. (18) did not state whether blinding was performed and were therefore assessed as unclear risk of bias. Other studies (13, 14, 19, 21, 22) described blinding of participants and personnel and blinding of outcome assessment, and were graded as low risk.

### Incomplete outcome data and selective outcome reporting

Groeger (21) and Gilli (22) did not report the information of whether there were withdrawals or exclusions, and were therefore assessed as having an unclear risk of bias. The other four RCTs (13, 14, 18–20) did not have incomplete outcome data, and were graded as low risk.

All studies reported outcomes assessed in the protocol and were therefore assessed as low risk of bias.

### Other bias

Akbarzadeh et al. (20) used a commercial formulation containing 12 strains of probiotic species plus fructooligosaccharides as prebiotics, which may have biased the outcomes. Other studies (13, 14, 18, 19, 21, 22) did not have other biases and were graded as low risk.

### Effects of probiotics on psoriasis severity

**Figure 4:**
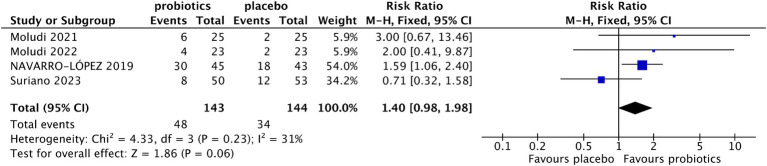
Forest plot of comparison between the probiotic and placebo groups for the percentage of patients with PASI⩾75% reduction.

Four studies reported the percentage of patients with ≥75% reduction from baseline in PASI (13, 18, 19, 21). The results indicated that more patients in the probiotic group achieved ≥75% reduction from baseline in PASI compared with the control group (33.57% vs. 23.61%), although the difference did not reach statistical significance (RR 1.40, 95% CI 0.98–1.98, *p* = 0.06) (Figure 4). The level of heterogeneity between the enrolled studies was not significant (I^2^ = 31%, *p* = 0.23). In the sensitivity analysis, when Suriano’s study (14) was excluded, the difference in the percentage of patients with ≥75% reduction from baseline in PASI between the two groups was significant (RR 1.75, 95% CI 1.18–2.60, *p* = 0.005; test of heterogeneity: I^2^ = 0, *p* = 0.69).

**Figure 5:**
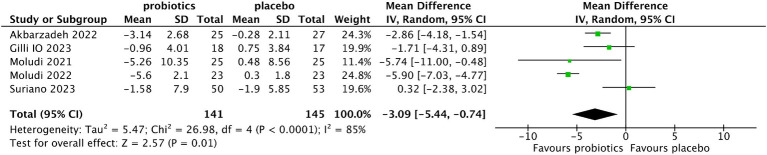
Forest plot of comparison between the probiotic and placebo groups for the reduction of PASI.

Five studies (13, 14, 19–21) evaluated the changes in PASI score from baseline after treatment. Compared with the control group, the reduction of PASI was more significant in the probiotic group (MD −3.09, 95% CI −5.04 to −0.74, *p* = 0.01; test of heterogeneity: I^2^ = 85%, *p* < 0.0001) (Figure 5). In the sensitivity analysis, when the study of Moludi (13) was deleted, the interstudy heterogeneity was eliminated and the results did not change (MD −2.08, 95% CI −3.91 to −0.24, *p* = 0.03; test of heterogeneity: I^2^ = 51%, *p* = 0.10). When the study of Akbarzadeh et al. (20) was deleted, the trend of a more significant reduction in PASI in the probiotic group remained unchanged, but the difference was not statistically significant (MD −3.16, 95% CI −6.61 to 0.30, *p* = 0.07; test of heterogeneity: I^2^ = 87%, *p* < 0.0001).

**Figure 6:**
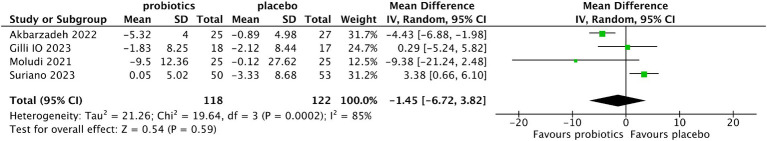
Forest plot of comparison between the probiotic and placebo groups for the reduction of DLQI.

Four studies (14, 19–21) reported the changes in DLQI from baseline after treatment. There was no significant difference in DLQI between the probiotic and placebo groups (MD −1.45, 95% CI −6.72 to 3.82, *p* = 0.59; test of heterogeneity: I^2^ = 85%, *p* = 0.0002) (Figure 6). Sensitivity analysis did not reveal any influence on the results when individual studies were excluded one by one, indicating that the results were stable.

### Effect of probiotics on inflammatory indicators

Serum levels of inflammation-related factors are often increased in patients with active psoriasis. Of the included studies, five evaluated changes in inflammatory indicators before and after treatment (13, 18, 19, 21, 22). Four studies investigated IL-6 (13, 18, 19, 21), 3 investigated CRP (13, 19, 21), 2 investigated TNF-α (18, 21), IL-1β (13, 18), and IL-23 (18, 22), and 1 study investigated IFN-γ (18), IL-17 (22), IL-12 (18), malondialdehyde (MDA) (19), total antioxidant capacity (TAC) (19), and LPS (13), respectively. Three studies showed that adjuvant treatment with single or mixed strains of *Bifidobacterium* and *Lactobacillus* significantly reduced the levels of serum inflammatory markers (CRP, IL-6, TNF-α, IL-1β, MDA and LPS) (13, 19, 21).

Raw data for the changes of mean difference before and after treatment were available for CRP in 3 studies (13, 19, 21) and for IL-6 in 2 studies (19, 21), making it possible to calculate the overall effects.

**Figure 7:**
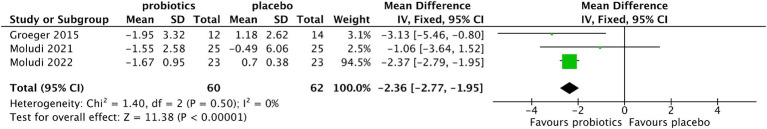
Forest plot of comparison between the probiotic and placebo groups for the serum level of CRP.

The results showed that compared with the control group, the pooled MD of CRP levels decreased in the probiotic group after treatment with no significant heterogeneity among the included studies (MD −2.36, 95% CI −2.77 to −1.95, *p* < 0.0001; test of heterogeneity test: I^2^ = 0%, *p* = 0.50) (Figure 7). In the sensitivity analysis, when individual studies were removed one by one, the results did not change.

**Figure 8:**
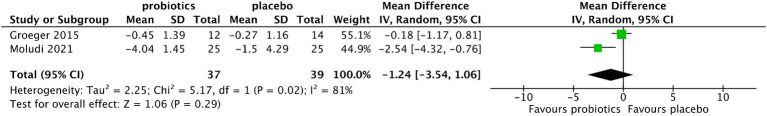
Forest plot of comparison between the probiotic and placebo groups for the serum level of IL-6.

Pooled analysis showed that the reduction of IL-6 in the probiotic group was not significant compared with that in the control group (MD −1.24, 95%CI −3.54 to 1.06, *p* = 0.29), and the level of heterogeneity among the included studies was significant (I^2^ = 81%, *p* = 0.02) (Figure 8).

### Subgroup analysis for treatment duration

Subgroup analyses were conducted for treatment duration, and the results were shown in Table 2.

**Table 2:** Subgroup analysis for treatment duration.

Treatment duration	No. of studies	Pooled estimate		*p*-value	Test of heterogeneity	
		Mean difference	95% CI		*I^2^* (%)	*p-*value
PASI						
4 weeks	1	−0.49	−1.93, 0.95	0.50	–	–
8 weeks*	4	−2.29	−2.69, −1.90	<0.00001	17	0.31
12 weeks	1	−2.86	−4.18, −1.54	<0.0001	–	–
6 months	1	0.32	−2.38, 3.02	0.82	–	–
Total	5	−3.09	−5.44, −0.74	0.01	85	<0.0001
DLQI						
4 weeks	1	−1.01	−3.68, 1.66	0.46	–	–
8 weeks*	3	−2.29	−4.56, −0.03	0.05	11	0.33
12 weeks	1	−4.43	−6.88, −1.98	0.0004	–	–
6 months	1	3.38	0.66, 6.10	0.01	–	–
Total	4	−1.45	−6.72, 3.82	0.59	85	0.0002

*Treatment duration of the study by Gilli et al. (22) was 60 days.

Only 1 study (20) reported PASI and DLQI at baseline and at week 4, comparing the change in the scores after treatment between the probiotic and placebo groups, which was not statistically significant (MD −0.49, 95% CI −1.93 to 0.95, *p* = 0.50).

At week 8 and 12, the PASI and DLQI decreased more significantly in the probiotic group than in the control group, with no heterogeneity among studies, suggesting that probiotics had a therapeutic effect on psoriasis lesions. However, the results from Suriano et al. (14) at 6 months showed no significant changes in PASI and DLQI from baseline between the probiotic and placebo groups (Table 2).

### Adverse events

**Figure 9:**
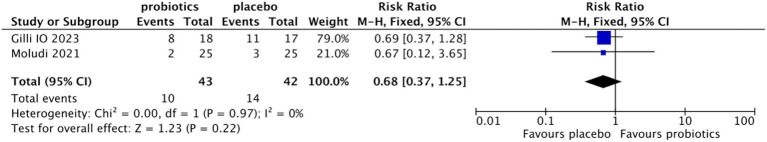
Forest plot of comparison between the probiotic group and the placebo group for adverse events.

Of all the studies included, only two reported the incidence of AEs (13, 22). The pooled rates of the probiotic and placebo groups were 23.26% and 33.33%, respectively. No significant difference was found between the two groups (RR 0.68, 95% CI 0.37–1.25, *p* = 0.22; test of heterogeneity: I^2^ = 0%, *p* = 0.97) (Figure 9). The symptoms reported were nausea, flatulence, diarrhea, and abdominal discomfort. No serious AEs occurred in both groups.

## Discussion

Psoriasis is a chronic relapsing inflammatory skin disease, and its pathogenic mechanisms are closely related to individual genetic susceptibility and environmental factors. The relationship between gut microbiota imbalance and psoriasis has attracted much attention. However, until now, there has been no direct evidence to confirm the direct causal relationship between gut microbiota imbalance and the onset and deterioration of psoriasis. Several recent Mendelian randomization studies have shown that certain gut microbiota is causally linked to psoriasis, with potential as diagnostic markers and targets for therapeutic intervention (23–26). A recent animal study indicated that the severity of psoriasis-like skin phenotype was accompanied by changes in the composition of the gut microbiota. Fecal microbiota transplantation from mice with severe psoriasis skin phenotype aggravated psoriasis skin inflammation in mildly symptomatic mice, accompanied by increased infiltration and differentiation of Th17 and changes in colon microbiota. These results provide evidence that gut microbiota may regulate host metabolism and psoriasis skin inflammation in mice (11). However, the only RCT with a small sample failed to confirm the effectiveness of fecal microbiota transplantation in active peripheral psoriatic arthritis (27). Recent studies have shown that targeted anti-cytokine therapy alleviates psoriasis with concomitant restoration of gut microbiota homeostasis (28, 29). Therefore, from the perspective of correcting the imbalance of gut microbiota in the treatment of psoriasis, the effectiveness of probiotics has attracted attention. However, to date, randomized, double-blind, placebo-controlled studies of oral probiotics for the treatment of psoriasis are limited and the results are inconsistent. A meta-analysis that included two RCTs showed that oral probiotics improved PASI in patients with psoriasis, but PASI was described differently in each study and could not be combined for statistical analysis (15). Furthermore, only 1 study compared changes in serum levels of inflammatory indicators before and after treatment (15). In our meta-analysis, more recently published RCTs were included and showed that PASI decreased more significantly from baseline in the probiotic group after treatment than in the placebo group. The percentage of patients with ≥75% reduction from baseline in PASI after treatment showed an increasing trend in the probiotic group compared with the placebo group. With the improvement in the clinical manifestations of psoriasis, the serum CRP level in the probiotic group decreased more significantly than that in the placebo group.

The mechanisms by which probiotics such as *Bifidobacterium* and *Lactobacillus* improve the severity of skin lesions and inflammation in psoriasis are unknown. Recent studies have shown that the microbiome in patients with psoriasis is significantly different from that in healthy controls (30). The diversity of intestinal microbiota decreases and the relative abundance of some beneficial bacteria such as *Lactobacillus*, *Bifidobacterium*, *Prevotella*, and *Akkermansia* reduces (7, 31). With a decrease in beneficial bacteria in the gut, the production of acid- and antibiotic-like compounds is reduced, and the ability to prevent pathogen invasion is affected (32, 33). Therefore, pathogens will produce endotoxins to destroy the intestinal mucosal barrier, leading to the activation of effector T cells and secretion of proinflammatory cytokines, causing local or systemic immune responses (34). Supplementation with probiotics can directly increase the abundance of beneficial bacteria in the gut and prevent the colonization of pathogens, thus reducing the damage of endotoxins to the intestinal mucosa and the production of proinflammatory cytokines (35). On the other hand, short chain fatty acids (SCFAs), as microbiota-derived fermentation products, also play a key role in promoting intestinal barrier integrity and exerting anti-inflammatory effects (36). SCFAs can regulate the number and function of T cell populations in the colon microenvironment through the Th17/IL-23 pathway, and restore the immune balance of Th1/Th2 and Th17/Treg (37). Patients with psoriasis have reduced SCFA-producing bacteria in their gut microbiota, such as *Bacteroidetes*, and *Faecallibacterium*, which may contribute to defects in Tregs (38). Probiotic supplementation can increase the content of SCFAs in the gut, thus promoting the differentiation of Treg cells and the secretion of anti-inflammatory cytokines to exert anti-inflammatory effects.

Psoriasis is a chronic recurrent disease; therefore, the safety of probiotic supplementation should be of great concern. Subgroup analysis for the duration of oral probiotics was performed in our study. In the study of Akbarzadeh et al. (20), there was no significant difference in the changes of PASI and DLQI in the experimental group compared with the placebo group after 4 weeks of probiotic treatment. At weeks 8 and 12, the pooled PASI and DLQI in the probiotic group declined significantly more than those in the placebo group. After treatment, the combined MDs of PASI and DLQI in the probiotic group had a more significant trend of decreasing with an increase in the treatment course than that in the placebo group. However, because of the small number of the included studies, the results need to be interpreted with caution. In the RCT study of Suriano et al. (14), the results of long-term oral administration of probiotics for 6 months did not support the above results. More RCTs are needed to determine the optimal course of probiotic supplementation and the relationship between the treatment duration and the efficacy. Regarding safety concerns, probiotics are generally believed to have beneficial effects on human health by shaping the gut microbiota. Most lactic acid-producing bacteria are non-pathogenic and non-toxic. The probiotics used in the included study were *Bifidobacterium* (13, 18, 21) or *Lactobacillus* (14, 19, 20, 22), or mixed genera with *Bifidobacterium* or *Lactobacillus* (20). However, only two studies reported the incidence of AEs between the two groups, and the results showed that there was no significant difference between the probiotic and placebo groups during treatment. The common AEs reported were mainly gastrointestinal reactions, including nausea, abdominal distension, abdominal discomfort, and diarrhea, etc., and no serious AEs occurred. Unfortunately, in the study of Suriano et al. (14), *Lactobacillus rhamnosus* was administered for up to 6 months, and no data on AEs were reported. Therefore, the use of probiotics in the treatment of psoriasis is generally safe; however, more RCTs are necessary to further evaluate the effective strains and their safety, especially for long-term use.

It should be noted that, of the included studies, two did not mention whether additional therapies were used, and five explicitly stated that patients received various local or systemic therapies simultaneously. Therefore, probiotics were only an adjunct to conventional treatment in these studies. Our findings suggest that, although the characteristics of the patients and the basic treatment varied in the included studies, the combination of probiotics in addition to conventional treatment could further improve the severity scores and inflammatory indicators of psoriasis. No randomized controlled studies have been reported in the treatment of psoriasis with probiotics alone.

The advantage of this meta-analysis was that we included several RCTs on probiotics in the treatment of psoriasis published in recent years, which updated the results of the previous meta-analysis and made the results more objective and convincing. The disadvantages of this study were as follows: First, the number of studies included in this meta-analysis was less than 10, which could not be analyzed for publication bias (17). In addition, the sample size was small (400 participants), which could have biased the results. Second, significant clinical heterogeneity existed in the included studies due to differences in initial disease severity, the scoring systems used to assess disease severity, and the strains and courses of probiotics used in individual studies. Despite the use of a random-effects model, the clinical heterogeneity of the studies could still lead to bias in the pooled results. In addition, in patients with mild psoriasis (PASI<10), the 75% reduction in PASI may not always accurately evaluate the improvement after treatment. Sensitivity analysis revealed that the percentage of patients with PASI reduced by 75% or more was more statistically significant in the probiotic group than in the control group after removing the study of Suriano (14). In the study of Suriano, although oral probiotics were taken for up to 6 months, the rates between the two groups were not statistically significantly different (14). Sensitivity analysis after removal of the study by Akbarzadeh et al. indicated that the changes in PASI from baseline after treatment became statistically insignificant in the probiotic group compared with the control group (20). In the study of Akbarzadeh et al., a prebiotic (fructooligosaccharide) was added to a mixture of 12 probiotic strains, which may have a synergistic effect with the probiotics, leading to overestimation of the effectiveness of the treatment (20). Third, probiotics are believed to have therapeutic effects in psoriasis by improving immune inflammation. Although five included studies (13, 18, 19, 21, 22) evaluated various inflammatory indicators before and after treatment, our study only compared the changes in CRP and IL-6 levels between the two groups, because raw data could not be extracted or the inflammatory indicators detected in individual studies were different. Previous studies showed that PASI was significantly associated with increased CRP level, especially in patients with moderate and severe psoriasis, and CRP level decreased with remission of psoriasis. In untreated psoriasis patients without arthritis, CRP can be interchanged with PASI as a measure of disease severity (39, 40). IL-6 is a pleiotropic proinflammatory cytokine that is elevated in serum and skin lesions in patients with psoriasis. However, inhibition of IL-6 may lead to compensatory proinflammatory effects of other cytokines making anti-IL-6 therapy ineffective for psoriasis (41). Although IL-6 is not the most important mediator of the inflammatory pathway in the skin environment, it is an interesting biomarker candidate for predicting psoriasis treatment response (42). However, due to the small number of included studies, the observed inflammatory indicators and the way they were described differed in individual studies, clinical heterogeneity was too significant to conduct a summarized analysis.

In conclusion, the results of our study indicated that patients with psoriasis treated with oral probiotics as an adjuvant therapy significantly decreased PASI compared with the placebo group, and the percentage of patients with PASI decreased by ≧75% after treatment showed an increasing trend. With the improvement in the clinical severity of psoriasis, the serum CRP level in the probiotic group decreased more significantly than that in the placebo group. These results suggest that oral probiotics may reduce psoriasis severity by regulating gut microbiota and decreasing immune inflammation caused by microbiotic imbalance. Further large randomized controlled trials are required to verify this conclusion.

## Data Availability

The original contributions presented in the study are included in the article/Supplementary material, further inquiries can be directed to the corresponding author.

## Footnotes

^1^ https://www.math.hkbu.edu.hk/~tongt/papers/median2mean.html
