# Supplementary material for: The efficacy and safety of probiotics in the adjuvant treatment of psoriasis: a systematic review and meta-analysis of randomized controlled trials
Source: Front Med (Lausanne). 2024 Sep 6;11:1448626. doi: 10.3389/fmed.2024.1448626 (PMC11426359; doi:10.3389/fmed.2024.1448626)
Supplement: Supplementary file 2 [file Table_2.DOCX]

Table S2 Quality assessment of all included studies (Cochrane).

| **Study**  **(RCT)** | | **Sequence generation** | **Allocation concealment** | **Blinding** | **Incomplete outcome data** | **Selective outcome reporting** | **Other sources of bias** | **Quality (score)** |
| --- | --- | --- | --- | --- | --- | --- | --- | --- |
| 2019 | Navarro-Lopez V | ★ |  |  | ★ | **★** | **★** | 4 |
| 2021 | Moludi J | ★ | ★ | ★ | ★ | ★ | **★** | 6 |
| 2022 | Moludi J | ★ | ★ | ★ | ★ | ★ | ★ | 6 |
| 2022 | Akbarzadeh A |  |  |  | ★ | ★ |  | 2 |
| 2013 | Groeger D |  |  | ★ |  | ★ | ★ | 3 |
| 2023 | Suriano ES | ★ | ★ | ★ | ★ | ★ | ★ | 6 |
| 2023 | Gilli IO |  |  | ★ |  | ★ | ★ | 3 |
